# Air transportation carbon dioxide emission forecasting: An improved back propagation neural network

**DOI:** 10.1371/journal.pone.0333226

**Published:** 2025-10-07

**Authors:** Peiwen Zhang, Yunan Luo, Qian Yu, Zhifeng Zhou

**Affiliations:** 1 School of Economics and Management, Civil Aviation Flight University of China, Guanghan, China; 2 Key Laboratory for Civil Aviation Data Goverance and Decision Optimization, Civil Aviation Management Institute of China, Beijing, China; 3 Chengdu Low altitude Economy High quality Development Research Center, Chengdu, China; Massey University, NEW ZEALAND

## Abstract

To address the challenges of increasing carbon dioxide (CO_2_) emissions and climate change caused by the growth of air traffic, accurate prediction of CO_2_ emissions in civil aviation has become crucial. This study proposes a CO_2_ emission prediction method based on an improved back propagation (BP) neural network, where the Improved Sparrow Search Algorithm (ISSA) is employed to optimize the hyperparameters of the BP neural network, thereby enhancing the prediction capability for CO_2_ emissions in civil aviation. To overcome the limitations of the traditional SSA, such as the tendency to fall into local optima during population initialization and the search process, this paper introduces Tent mapping for population initialization and incorporates adaptive t-distribution-based perturbation for individual position updates during the mutation operation, aiming to improve the algorithm’s global search ability and convergence performance. Subsequently, the ISSA algorithm is applied to optimize the weights and biases of the BP neural network, further constructing an ISSA-BP neural network-based prediction model for civil aviation CO_2_ emissions. Experimental results demonstrate that the improved BP neural network outperforms other comparative models in terms of prediction accuracy and error control, enabling accurate prediction of civil aviation CO_2_ emissions. This research provides a solid theoretical foundation for formulating precise energy-saving and emission-reduction strategies in civil aviation.

## 1. Introduction

Greenhouse gas emissions are causing global climate change. This is a focus of international relations and one of the most severe challenges facing human society today [1]. To address this challenge, countries have entered various global agreements aimed at collectively reducing greenhouse gas emissions.

Air transportation plays an indispensable role in both international and domestic trade, significantly contributing to economic development [2]. However, the expansion of the aviation industry has also posed formidable challenges for environmental governance. The sector has become one of the fastest-growing sources of greenhouse gas emissions, with an annual growth rate of 3.4% from 2010 to 2019 [3]. Recent IATA reports indicate a strong post-pandemic recovery, with global RPK (measured in Revenue Passenger Kilometers, RPK, a standard metric representing one paying passenger transported one kilometer) nearly returning to pre-pandemic levels and projected to grow steadily at around 3.75% annually over the next two decades [4]. This growth trajectory underscores the urgency of addressing aviation emissions through robust forecasting tools that account for both economic demand dynamics and exogenous shocks [5].

Therefore, without effective emission reduction measures, the aviation industry’s negative impact on climate change will continue to intensify. Reducing carbon emissions from the aviation industry is of paramount practical significance for controlling global carbon emissions and mitigating climate change.

In recent years, numerous scholars have begun to conduct in-depth research on carbon emissions across various fields. A review of recent studies reveals that research on carbon emissions in civil aviation primarily focuses on the following aspects: carbon emission accounting, identification of influencing factors, carbon reduction potential, and carbon emission trend prediction. For instance, Zhu et al. [6] proposed dividing the flight process into key phases such as takeoff, landing, climb, cruise, and descent to improve the accuracy of civil aviation carbon emission accounting, systematically calculating the carbon emissions for each phase. Liu et al. [7] based on phase efficiency evaluation, applied index decomposition analysis and attribution analysis to decompose carbon emission changes of 15 global airlines into seven influencing factors, and further explored the impact of each factor on carbon emission changes. Minami Kito [8] focused on the role of green aircraft with higher fuel efficiency in emission reduction, revealing the limitations of green technology applications. The results indicated that relying solely on the introduction of more fuel-efficient green aircraft is insufficient to achieve effective carbon reduction. Xu et al. [9] developed a bottom-up model of China’s civil aviation energy system based on the LEAP (Long-range Energy Alternatives Planning) model, an integrated energy-environment modeling tool, and analyzed the driving factors and development trends of the civil aviation industry under different scenarios. While valuable, such approaches lack the granularity to capture nonlinear relationships in aviation emissions, a challenge increasingly tackled by machine learning methods [10].

Accurate estimation and prediction of CO_2_ emissions are crucial for formulating effective energy-saving and emission-reduction policies and exploring future CO_2_ reduction models [11]. The accuracy of these predictions not only affects the timeline of emission reduction plans but also influences the successful implementation of specific mitigation measures. As Aldy [12] demonstrates, traditional econometric methods remain benchmarks for emissions forecasting, but their rigidity in handling hierarchical data structures limits their adaptability—a gap that neural networks can address [13].

Currently, the primary methods for predicting carbon emissions in civil aviation include Monte Carlo simulation, regression analysis, the STIRPAT model, the LEAP model, system dynamics models, and neural network models.

These traditional prediction methods have been widely applied in carbon emission forecasting. For instance, Liu et al. [14] utilized data from 1985 to 2015 to conduct scenario-based predictions of CO_2_ emissions from 2016 to 2030 using Monte Carlo simulation. Similarly, Chao et al. [15] employed life cycle assessment and Monte Carlo simulation to analyze the impact of sustainable aviation fuel (SAF) on emissions under different policy scenarios. Yu et al. [16] combined multiple linear regression with scenario analysis to establish eight different scenarios for predicting carbon emission trends in China’s civil aviation industry. Yang et al. [17] proposed a two-layer bottom-up emission prediction method based on the Autoregressive Integrated Moving Average (ARIMA) model by forecasting carbon emissions from round-trip flights in Shanghai.

These traditional methods have been widely adopted due to their simplicity, but they struggle with nonlinearities and endogenous relationships. For example, Brons et al. meta-analysis shows that aviation demand elasticities vary significantly across regions and trip types, complicating linear modeling [18]. Moreover these methods exhibit certain limitations when dealing with complex nonlinear problems, such as: difficulty in capturing intricate dependencies in emission data; limited adaptability to dynamic changes in aviation operations. These limitations become particularly critical in aviation emission forecasting, where the complex interplay of multiple factors creates highly nonlinear relationships that traditional linear models struggle to represent adequately. Consequently, in recent years, artificial intelligence, particularly neural network approaches, has gradually become a vital tool in the field of carbon emission prediction. For example, Yang et al. [19] combined Monte Carlo simulation with a Backpropagation (BP) neural network to predict CO_2_ emissions from China’s civil aviation transportation, demonstrating that this method could provide strong support for achieving carbon peak and carbon neutrality goals in China’s aviation industry. Wang et al. [20] proposed two hybrid models, ARIMA-BPNN and BPNN-ARIMA, by integrating the ARIMA method with a BP neural network (BPNN), and simulated carbon emissions for China, India, the United States, and the European Union under a no-pandemic scenario. The results showed that the average relative error of the predictions was approximately 1%, indicating high simulation accuracy. Zhao et al. [21] employed a mixed-data sampling approach with a Backpropagation neural network (MIDAS-BP model) for CO_2_ emission prediction, demonstrating that the model is suitable for both short-term and long-term CO_2_ emission forecasting. And these studies rarely address causal identification—a key requirement for policy analysis, as highlighted by Deschênes and Greenstone in their instrumental variables approach to environmental outcomes [22].

In summary, BP neural networks demonstrate strong performance in prediction tasks; however, traditional BP neural networks often suffer from getting trapped in local minima during optimization and exhibit slow convergence rates. To address these issues and achieve more accurate prediction results, this paper proposes a CO_2_ emission prediction method based on an improved BP neural network. This method enhances the SSA algorithm to effectively optimize the hyperparameters of the BP neural network, thereby improving prediction accuracy for carbon emissions in civil aviation.

The remainder of this paper is organized as follows: Section 2 introduces the ISSA-BP neural network model and its prediction process. Section 3 presents the data sources and empirical results comparing ISSA-BP with other models using 1985–2019 aviation CO_2_ emission data. Section 4 concludes the study.

This study makes the following contributions:

(1) An ISSA is proposed, which integrates tent mapping and adaptive t-distribution mutation to increase the performance of the SSA. Compared with the traditional SSA, the ISSA effectively mitigates randomness in population initialization and has a significantly enhanced capacity to escape local optima during iterations. These advancements provide considerable improvements in global search capabilities and convergence speed.
(2) A novel method for predicting carbon emissions in civil aviation, grounded in the ISSA-BP neural network framework, is introduced. To address the challenges of sensitivity and overfitting typically associated with BP neural networks, this study employs the ISSA to optimize the weight and bias parameters, thereby substantially improving the model’s robustness and predictive accuracy. Compared with conventional BP neural network optimization techniques, the proposed ISSA-BP approach demonstrates superior accuracy and stability in forecasting civil aviation CO_2_ emissions.
(3) The proposed CO_2_ emission prediction method for civil aviation based on the ISSA-BP neural network not only outperforms various existing models in terms of accuracy but also provides a robust theoretical foundation for relevant authorities. This foundation assists in the formulation of timelines for emission reduction strategies and the implementation of corresponding measures. This study offers new insights for enhancing CO_2_ emission prediction methods in civil aviation, with the potential to significantly influence the development of future emission reduction policies.

## 2. Methodology

### 2.1. Back propagation (BP) neural network

**Fig 1:**
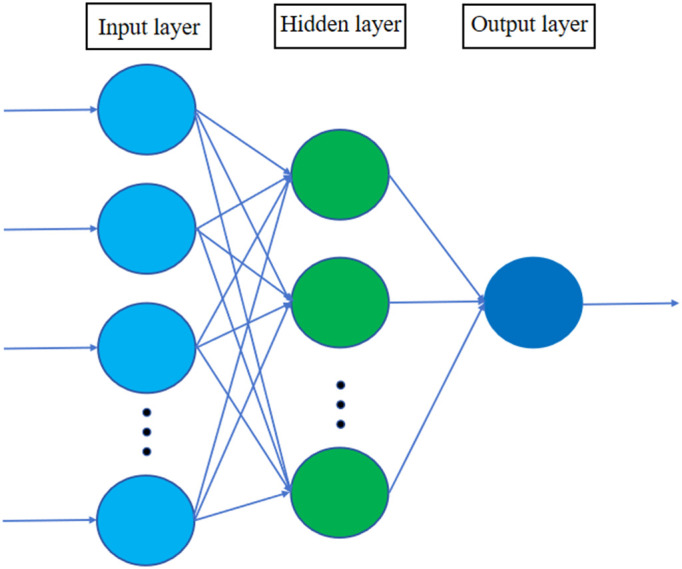
Schematic diagram of the BP structure.

The BP neural network, a widely used multilayer feed forward neural network for addressing complex and nonlinear prediction problems, was first proposed by Rumelhart and Geoffrey Hinton in 1986 [23]. This network employs the error back propagation algorithm for training, enabling it to achieve nonlinear mapping from input to output [24,25]. A three-layer BP neural network has been demonstrated to effectively handle various nonlinear information [26], exhibiting characteristics such as straightforward computation, robust nonlinear mapping capabilities, strong generalization ability, and scalability, as illustrated in Fig 1.

A BP neural network includes an input layer, a hidden layer, and an output layer. Its information processing is divided into forward propagation of data and backward propagation of errors to align the network output with the expected results.

The prediction principle of a BP neural network can be expressed by Eq. (1):


$$y_{j}=f(\sum_{i=1}^{n}\;w_{ji}x_{i}+b_{j})$$ (1)

where *y*_*j*_ represents the output of the network, *f* (^.^) represents the activation function, *w*_*ji*_ represents the connection weight between the *j*^-th^ neuron and the *i*^-th^ neuron, *x*_*i*_ represents the input variable, and *b*_*j*_ represents the bias value of the *j*-th neuron.

The specific procedure of the BP neural network consists of the following steps:

(1) Determination of the network topology structure: The number of hidden layer nodes is determined on the basis of the golden section method (an efficient search algorithm based on the golden ratio) and the principle of minimum error (selecting the configuration that yields the smallest prediction error on validation data), as expressed in Eq. (2). Specifically, several nodes are established for learning. Then, nodes are added one by one, and finally, the final number of nodes in the middle hidden layer is determined. The formula for the number of hidden layer nodes *n* is as follows:

$$n=\sqrt{l+m}+a$$ (2)

where *l* represents the number of input layers, *m* represents the number of output layers, and *a* is an integer between 1 and 10.

(2) Network initialization: The weights and biases between the input layer, hidden layer, and output layer, with initial values randomly generated. Flatten all weights and biases to be optimized in BP neural network into a vector, as expressed in Eq. (3).

$$\theta ={\left[\;\;\begin{array}{c} {}*20cw_{11}^{\left(1\right)},w_{12}^{\left(1\right)},\ldots ,w_{1d}^{\left(1\right)},\ldots ,w_{md}^{\left(L\right)},b_{1}^{\left(1\right)},\ldots ,b_{n}^{\left(L\right)} \end{array}\;\;\;\right]}^{⊤}$$ (3)

where contains all connection weights *w* from input layer to hidden layer, hidden layer to output layer, and bias *b* of each neuron. Each parameter component is searched in the unified interval [− 1], which ensures the diversity of initial solutions and is not easy to cross the boundary.

(3) Data preprocessing: The data are randomly divided into training and test sets. For the convenience of training, it is necessary to normalize each data point to eliminate the influence of different dimensions on the results. This approach was preferred over alternatives like z-score normalization because: it preserves relative relationships in our time-series emission data while eliminating scale differences; it optimally matches the BP neural network’s sigmoid activation functions that inherently operate in [0,1] ranges; and it provides intuitive interpretability as normalized values directly reflect percentage changes in emission levels. Therefore, in this study, the normalization method expressed by Eq. (4) is applied as follows:

$$x_{i}^{*}=\frac{x_{i}-x_{min}}{x_{max}-x_{min}}$$ (4)

where x_i_^*^ denotes the normalized data; *x*_*i*_ represents the sample observation value of the corresponding variable; and *x*_min_ and *x*_max_ are the minimum and maximum values, respectively, of the sample of the corresponding variable.

(4) Data training and testing: On the basis of the determined network structure, the network is trained on the training set, and simulation testing is conducted on the test set to verify the effectiveness of the model.

### 2.2. Improved sparrow search algorithm (ISSA)

In recent years, there has been a growing trend in employing swarm intelligence optimization algorithms for parameter optimization. These algorithms are celebrated for their strong adaptability, high robustness, and excellent scalability. Notable examples include the SSA, grey wolf optimization (GWO) algorithm, whale optimization algorithm (WOA), and particle swarm optimization (PSO) algorithm.

Compared with GWO and PSO, the SSA offers advantages such as fewer parameters, faster convergence, simpler computations, and easier implementation [27]. However, a notable drawback of the SSA is its tendency to diminish population diversity as it approaches the globally optimal solution, often resulting in convergence to local optima. To address this limitation, numerous researchers have proposed improvements to the algorithm. For example, Yang et al. [28] utilized Chaotic mapping to initialize the sparrow population, thereby overcoming the issues associated with random initialization and bolstering the foundation for global search. Wang et al. [29] applied an adaptive t-distribution mutation strategy to enhance the SSA, enabling the algorithm to escape local extremes.

In this paper, we advance the SSA by integrating adaptive t-distribution mutation and employing tent mapping for the initialization of the sparrow population (hereafter referred to as ISSA, Improved Sparrow Search Algorithm). This dual approach is designed to augment the algorithm’s global search capabilities and enhance its convergence performance.

A common challenge in population-based optimization is premature convergence to local optima, where individuals cluster around suboptimal peaks and stall. In ISSA, this is mitigated by two mechanisms: (1) Tent map initialization generates a more uniform initial population in the whole search space to prevent early convergence; (2) The adaptive t-distribution mutation generates large and heavy tailed disturbances at the initial stage of iteration, jumps out of the shallow trap, and then converges to fine search at the later stage, dynamically balancing global exploration and local development. The synergy of these two improvements can significantly reduce the stagnation risk of SSA in multimodal problems and improve the robustness of the algorithm.

#### 2.2.1. Sparrow search algorithm (SSA).

The SSA, introduced in 2020 by Xue and Shen, is a novel swarm intelligence optimization method inspired by the foraging and antipredation behaviors of sparrows [27]. This algorithm categorizes the population into three distinct roles: finders, followers, and alerters. Finders are tasked with locating food and guiding the group, while followers closely trail them, and alerters monitor the environment for potential dangers. The optimization process in the SSA mirrors the behaviors associated with these roles.

In the SSA, the objective function represents food, and the variable corresponds to the positions of the sparrows. The locations of the finders are updated according to Eq. (5):


$$X_{i,j}^{t+1}=\{\begin{array}{c} {}*20cX_{i,j}^{t}\exp\left(\frac{-i}{\alpha iter_{\max}}\right),R_{2}<ST\\ X_{i,j}^{t}+QL,R_{2}\geq ST \end{array}$$ (5)

where *t* denotes the current iteration; *j* = (1, 2,..., *d*); *X_i,j_^t + 1^* indicates the position information of the *i*^-th^ sparrow in the j^-th^ dimension; *iter*_max_ represents the maximum number of iterations; *α* ∈(0, 1), where *α* is a randomly generated number within the range of −1–1; *R*_2_ and *ST* denote the warning value and safety value, respectively; *Q* indicates a normally distributed random number; and L is a 1 × d matrix with unit internal elements. When *R*_2_ *<* *ST*, the area is safe, and the finders conduct extensive search patterns. When *R*_2_* ≥* *ST*, some sparrows have detected natural enemies, and the entire population needs to move to a safe area as soon as possible.

Followers follow finders to search for food. The follower locations are updated according to Eq. (6):


$$X_{i,j}^{t+1}=\{\begin{array}{c} {}*20cQexp\left(\frac{X_{worst}^{t}-X_{i,j}^{t}}{t^{2}}\right),i>\frac{N}{\text{2}}\\ X_{p}^{t+1}+\left|X_{i,j}^{t}-X_{p}^{t+1}\right|\times A^{+}\times L,other \end{array}$$ (6)

where *N* refers to the size of the sparrow population, *X*_*P*_ denotes the best positions of the finders, *X^t^*_worst_ represents the current worst positions, and *A* is a binary 1 × d matrix with randomly assigned internal elements of either 1 or −1. When *i* > *N*/2, the *i*^-th^ follower with the worst fitness value is most likely to be hungry. Its energy value is low, and it needs to search for food in other areas to replenish its energy.

The alerters are responsible for providing safety warnings. The alerter location is updated via Eq. (7):


$$X_{i,j}^{t+1}=\{\begin{array}{c} {}*20cX_{best}^{t}+b\times \left|X_{i,j}^{t}-X_{best}^{t}\right|,f_{i}>f_{g}\\ X_{i,j}^{t}+K\times \left(\frac{\left|X_{i,j}^{t}-X_{worst}^{t}\right|}{\left(f_{i}-f_{w}\right)+e}\right),f_{i}=f_{g} \end{array}$$ (7)

where *Xt best* represents the current optimal position and *b* is a normal distribution of random numbers and is considered a step size control parameter. Moreover, K is a random number varying within the range of [−1, 1], *f*_*i*_ denotes the fitness of an individual sparrow, and *f*_*g*_ and *f*_*w*_ represent the best and worst fitness, respectively, of the current position. Finally, *e* is a small arbitrary number used to avoid zeros in the denominator.

In short, sparrow populations iterate according to the rules of Eqs. (5) to (7), constantly updating their positions to find lower-risk food until the conditions are met.

#### 2.2.2. Improvement strategy.

The SSA may lead to an uneven distribution of the population in the search space because of the use of random population initialization, thus weakening the global search ability. To improve the diversity and uniformity of the population, in this work, tent mapping is used for population initialization. In addition, the SSA may fall into local optimality due to the existence of multiple local minima in the objective function during the optimization process. To improve the global search accuracy, here, adopts adaptive t-distribution mutation is adopted to perturb the individual positions.

(1) Tent mapping

The application of the Tent map in optimization problems can not only effectively maintain population diversity but also assist the algorithm in escaping local optima, thereby enhancing global search capabilities.

The Tent map is defined as:


$$x_{n+1}=\{\begin{array}{c} {}*20crx_{n},0\leq x_{n}<0.5\\ r(1-x_{n}),0.5\leq x_{n}<1 \end{array}$$ (8)

In Eq. (8), *x*ₙ** represents the value at the *n*^-th^ iteration, and *r* is the parameter controlling system behavior, typically ranging between [0,1]. The mapping function manifests as a combination of two linear functions, exhibiting distinct nonlinear characteristics. The Tent map demonstrates different dynamic behaviors corresponding to varying parameter r values.

(2) Adaptive t-distribution mutation

The t-distribution, also known as the Student’s t-distribution, has a probability density function with *m* degrees of freedom:


$$p\left(x\right)=\frac{G\left(\frac{m+1}{\text{2}}\right)}{\sqrt{mp}G\left(\frac{m}{\text{2}}\right)}{\left(1+\frac{x^{2}}{\text{2}}\right)}^{-\frac{m+1}{\text{2}}}$$ (9)

In Eq. (9), $G\left(\frac{m+1}{\text{2}}\right)=\int_{0}^{+\infty }\;x^{\frac{m+1}{\text{2}}-1}e^{-x}dx$ is Euler integral of the second type, *m* affects the shape of the curve, which is specifically manifested as follows:


t(m→1)=C(0,1),t(m→∞)→N(0,1) (10)

In Eq. (10), C (0,1) is a Cauchy distribution, and N (0,1) is a Gaussian distribution. The t distribution combines the advantages of both the Cauchy and Gaussian distributions. The Cauchy distribution is effective in maintaining population diversity because of its strong global search capabilities, whereas the Gaussian distribution excels in local exploration, thereby accelerating the convergence speed [30].

To further enhance the optimization performance of the SSA and prevent the algorithm from falling into local optima during later iterations, an adaptive t-distribution mutation operator is employed. The degree of freedom parameter for this operator is defined by the number of iterations. The specific formula for this mutation is as follows:


$$X_{i}^{t+1}=X_{i}^{t}+X_{i}^{t}t\left(iter\right)$$ (11)

In Eq. (11), *X _i_^t + 1^* is the position of the perturbed sparrow *i*, and *X_i_^t^* is the position of sparrow *i* at the *t*^-th^ iteration. The proposed update adds a random interference term *X_i_^t^t*(*iter*) based on *X_i_^t^*, which not only utilizes the information on the current population but also introduces randomness. In the early stages of iteration, the t-distribution for mutation is similar to the Cauchy distribution, enhancing global exploration capabilities. In the later stage of iteration, the t-distribution for mutation tends toward a Gaussian distribution, which helps improve the local development ability and convergence speed.

### 2.3. Procedure for forecasting civil aviation CO_2_ emissions via the ISSA-BP model

**Fig 2:**
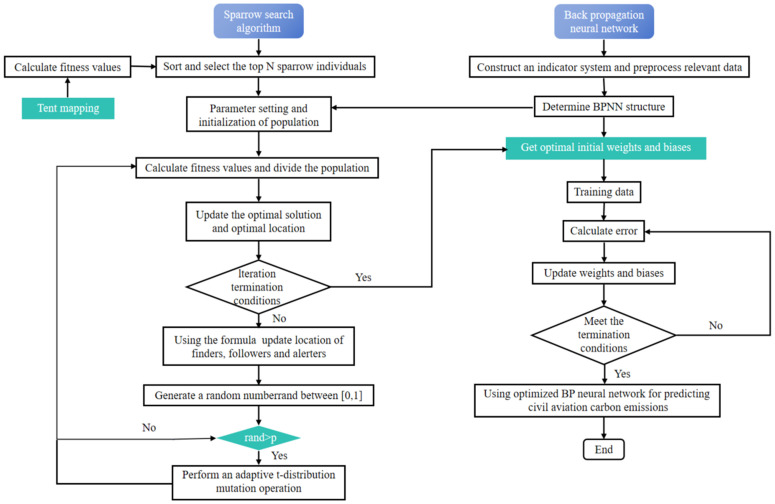
Process for predicting civil aviation CO_2_ emissions via the ISSA-BP model.

To accurately predict civil aviation CO_2_ emissions, the optimal weights and biases of the BP neural network are obtained through the ISSA. The specific steps for the ISSA-BP civil aviation carbon emission prediction model are outlined as follows, as illustrated in Fig 2.

(1) Construction of an Indicator System: On the basis of the relevant literature [14,16,19,31] and the specific context of civil aviation, the input indicators identified include transportation revenue, total transport turnover, total transportation volume, and energy intensity.
(2) Data Preprocessing: The input and output indicators are normalized via Equation (3), and the dataset is split into training and test sets, with 80% of the data allocated for training and the remaining 20% for testing.
(3) Determining the Topological Structure: The number of input layer nodes is set to 4, corresponding to the number of chosen indicators. The output layer contains 1 node, reflecting energy consumption. The number of hidden layer nodes is set to 13, resulting in a final configuration of 4-13-1.
(4) Population Initialization: The population is initialized via tent mapping to ensure diversity.
(5) Fitness Value Calculation and Population Division: The fitness value is computed for each sparrow, and the sparrows are arranged in descending order of fitness value. The top 20% are classified as finders, and the remainder are classified as followers.
(6) Updating the Optimal Solution and Position: The optimal solution and position are updated on the basis of the fitness scores.
(7) Checking the Iteration Limit: Whether the maximum number of iterations has been reached is determined. If so, the optimal weights and biases are output; otherwise, the next step is performed.
(8) Updating the Positions of the Sparrows: The positions of the finders, followers, and alerters are updated according to Equations (4)–(6).
(9) Random Number Generation: A random number rand is generated between [0, 1].
(10) Adaptive Mutation Decision: If rand>p is satisfied, the adaptive t-distribution mutation is performed; otherwise, return to step (4).
(11) BP Neural Network Training: The BP neural network is trained using the optimized parameters obtained.
(12) Output Error Calculation: The mean square error (MSE) is used as the error function to calculate the network’s output error.
(13) Updating Weights and Biases: The BP model parameters are adjusted on the basis of the calculated error.
(14) Checking the Convergence Conditions: If the convergence conditions are met, then the next step is performed; otherwise, return to step (12).
(15) Predicting CO_2_ Emissions: The optimized BP model is utilized to predict civil aviation CO_2_ emissions.

In summary, optimizing the BP neural network via the ISSA aims to enhance the model’s convergence performance and global search capabilities, significantly improving the accuracy and stability of CO_2_ emission predictions.

## 3. Data description and empirical comparison results

### 3.1. Data description

This study applied the idea of Zhou et al. [32] to calculate the CO_2_ emissions of China’s civil aviation transportation industry. Since aviation kerosene is the main fuel for civil aviation, this study assumes that all civil aviation CO_2_ emissions result from the combustion of jet kerosene. Using a “top-down” or “fuel-based” method, the total CO_2_ emissions of the civil aviation transportation industry are calculated by multiplying the fuel consumption by the CO_2_ emission coefficient for each fuel type and summing the results, as expressed in Eq. (12):


$$C=\sum_{i}\;EC_{i}\times HV_{i}\times EF_{i}$$ (12)

where *C* is the total emission of CO_2_ (unit: ton), *EC*_*i*_ is the fuel consumption of type *i* fuel (unit: ton), *HV*_*i*_ is the net calorific value of type *i* fuel (unit: GJ/ton), and *EF*_*i*_ is the emission coefficient of type *i* fuel (unit: ton/GJ).

**Fig 3:**
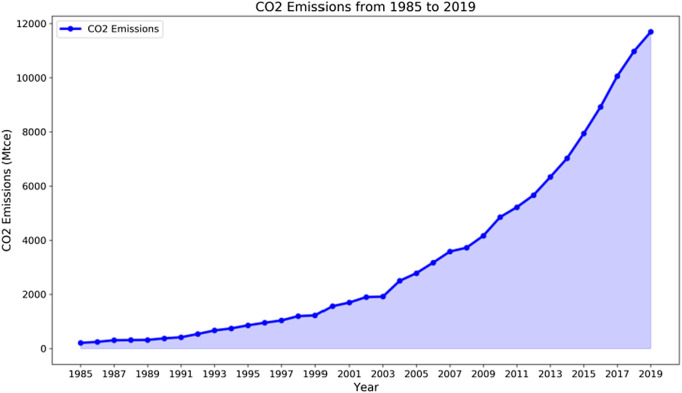
CO_2_ Emissions from 1985–2019.

This formula is used to calculate the historical carbon emission value of China’s civil aviation industry, as shown in Fig 3. According to the IPCC report, the net caloric value of jet kerosene is 44.1 GJ/ton, and the emission coefficient is 71.9 kg/GJ [33].

Data for all the study indices, including energy consumption, transportation revenue, total transportation turnover, total transportation volume, and energy intensity, are collected from the Statistical Data of the Civil Aviation of China and the China Civil Aviation Compendium of Statistics. Table 1 provides statistical descriptions of the variables. Transportation revenue represents the market and demand, whereas transportation turnover and volume reflect the transportation capacity and development scale of China’s civil aviation. Each unit turnover refers to energy intensity, which reflects the technological level.

**Table 1:** Statistical descriptions of the different civil aviation variables.

Variable	Symbol	Unit	Mean	Std. D.	Max.	Min.
Carbon emissions	C	Million tons	32.8934	33.0885	116.9476	2.0922
Energy consumption	E	Million tons	10.3767	10.4380	36.8920	0.6600
Transportation revenue	Y	100 million yuan	16.8251	18.0598	60.1837	0.1851
Total transport turnover	R	10,000 tkm	3385473.057	3688900.019	12932530.00	127102.00
Total transportation volume	Q	10,000 tons	1876.7913	1902.6927	6693.1400	86.7359
Energy intensity	EI	10, 000 ton/10,000 tkm	0.3647	0.0689	0.5193	0.2843

### 3.2. Evaluation index

Five indices are used to evaluate the prediction performance of the model, namely, the R-square determination coefficient (R^2^), mean absolute error (MAE), root mean squared error (RMSE), mean squared error (MSE), and mean absolute percentage error (MAPE) [34–36], which are expressed by the following equations:


$$R^{2}=1-\frac{\sum {\mathrm{\backslash nolimits}}_{i=1}^{n}\;{\left(y_{i}-{\hat{y}}_{i}\right)}^{2}}{\sum {\mathrm{\backslash nolimits}}_{i=1}^{n}\;{\left(y_{i}-\bar{y}\right)}^{2}}$$ (13)

$$MAE=\frac{\text{1}}{n}\sum_{i=1}^{n}\;\left|{\hat{y}}_{i}-y_{i}\right|$$ (14)

$$RMSE=\sqrt{\frac{\text{1}}{n}\sum_{i=1}^{n}\;{\left({\hat{y}}_{i}-y_{i}\right)}^{2}}$$ (15)

$$MSE=\frac{\text{1}}{n}\sum_{i=1}^{n}\;{\left({\hat{y}}_{i}-y_{i}\right)}^{2}$$ (16)

$$MAPE=\frac{\text{1}}{n}\sum_{i=1}^{n}\;\left|\frac{{\hat{y}}_{i}-y_{i}}{y_{i}}\right|\times 100\%$$ (17)

where *n* is the number of samples, *y*_*i*_ is the true value, ${\hat{y}}_{i}$ is the predicted value, and $\bar{y}$ is the average of all the true values.

A high R^2^ shows the model explains most variance, but can hide large individual errors; MAE gives the average error in tons of CO_2_; RMSE penalizes large deviations more heavily, important when overshoots may trigger quota breaches; MSE is its squared form; and MAPE expresses errors as a percentage of actual emissions, useful for relative‐target settings. Together, they ensure that both typical and extreme prediction errors are visible and properly interpreted in a policy context.

### 3.3. Correlation analysis

The correlation analysis between CO_2_ emissions of transportation revenue, total civil aviation turnover, total civil aviation volume, and energy intensity variables is carried out. The calculation results are shown in Table 2.

**Table 2:** Correlation test.

	C	Y	R	Q	EI
C	1	0.992**	0.999**	0.999**	−0.785**
Y	–	1	0.994**	0.993**	−0.806**
R	–	–	1	0.998**	−0.775**
Q	–	–	–	1	−0.790**
EI					1

Note: * * at level 0.01 (double tailed), the correlation is significant.

The regression results showed that all coefficients were significant at 1% level.

The correlation analysis results show that the correlation coefficients between variables *C, Y, R, Q* and *EI* are between 0.775 and 1.000, showing a significant correlation feature. This result suggests that there may be multicollinearity between the above variables, which needs further verification to avoid the model estimation bias.

In order to further verify the multicollinearity problem, this paper uses the variance inflation factor (VIF) for diagnostic analysis. Table 3 shows that the VIF values of all variables are higher than 10, which is significantly higher than the empirical standard (usually 5 or 10), indicating that there is a serious multicollinearity problem between variables, which may have a significant impact on the reliability of the model estimation results.

**Table 3:** Multiple collinearity diagnosis results.

	VIF
*Y*	106.2
*R*	541.5
*Q*	434.2
*EI*	4.311

For the multicollinearity problem, ridge regression method is used to estimate. As a regression technique specially dealing with multicollinearity, ridge regression effectively reduces the variance of regression coefficient by introducing L2 regularization term, thus alleviating the negative impact of collinearity on the stability of the model. Although ridge regression is a biased estimation method, which will lose some estimation accuracy compared with the least square method (OLS), it has significant advantages in improving the robustness of the model. See Table 4 for specific analysis results.

**Table 4:** Ridge regression analysis results.

Variable	B	SE(B)	Beta	B/SE(B)	R^2^	Adj R^2^	F	K (Ridge parameter)	p
ln*Y*	0.159	0.006	0.222	26.322	0.990	0.977	358.990	0.400	0.000
ln*R*	0.219	0.007	0.251	31.349					
ln*Q*	0.234	0.007	0.244	32.932					
ln*EI*	−1.252	0.075	−0.190	−16.728					
Constant	−3.436	0.173	0.000	−19.903					

The significance test results of the model showed that the statistical value F was 358.990, and the corresponding significance level p was 0.000 (p < 0.05), indicating that the regression model was statistically significant. Based on the regression results in Table 4, the multiple linear regression model constructed in this paper is shown in equation (18).


lnC=0.159lnY+0.219lnR+0.234lnQ−1.252lnEI−3.436 (18)

Through the analysis of the regression results, it can be seen that transportation revenue, total civil aviation turnover and total civil aviation volume are the main factors to promote the growth of CO_2_ emissions, while energy intensity has a significant negative impact on the growth of CO_2_ emissions. In addition, energy consumption intensity is considered to be the key factor affecting CO_2_ emissions. Specifically, a 1% change in energy intensity will lead to a 1.252% change in CO_2_ emissions.

### 3.4. Comparative optimization performance analysis of the ISSA

The original Sparrow Search Algorithm (SSA) suffers from common swarm intelligence limitations including premature convergence, slow convergence speed, and low accuracy. To address these issues, researchers have developed improved versions such as hybrid SSA [37], chaotic SSA [38], and multi-group co-evolution SSA [39], all demonstrating superior convergence speed and optimization performance compared to basic SSA. Therefore, to enhance global search capability and convergence performance, we improve SSA by incorporating adaptive t-distribution mutation and tent mapping for the initialization of the sparrow population.

BP neural networks frequently encounter challenges such as entrapment in local minima during the optimization process and exhibit sluggish convergence rates. To address these limitations and achieve more precise prediction results, this study introduces a novel CO_2_ emissions prediction method grounded in an improved BP neural network. This approach employs an improved SSA to optimize the hyperparameters of the BP neural network effectively.

The parameter settings of the ISSA are shown in Table 5.

**Table 5:** Parameter indicators of the ISSA.

Parameter indicators	Explanation	Value
POP	Sparrow population size	50
Max_iter	Maximum number of iterations	100
PD	Proportion of finders	0.2
SD	Proportion of alerters	0.2
ST	Warning value	0.8
p	Probability of adaptive t-distribution mutation	0.5
Random_state	Random number seed	1

The sparrow population size is set to 50, with a maximum number of iterations of 100. The proportions of finders and alerters are set to 20%, the warning value is 0.8, and the probability of adaptive t-distribution mutation is 0.5. To maintain experimental consistency, a fixed random number seed of 1 is utilized.

To evaluate the optimization performance of the ISSA, six benchmark test functions are selected, encompassing both unimodal and multimodal categories, as detailed in Table 6. By using benchmark functions of multiple categories, the optimization ability of the ISSA can be comprehensively evaluated. The study compares the ISSA with five other optimization algorithms: the SSA, GWO, PSO, the WOA, and ABC(Artificial Bee Colony). The parameter settings for all the algorithms are summarized in Table 7. Each test function undergoes twenty experimental iterations.

**Table 6:** Benchmark functions.

Unimodal function	Dimension	Upper and lower bounds	Optimal solution
$F_{1}\left(x\right)=\sum_{i=1}^{n}\;\left|x_{i}\right|+\prod_{i=1}^{n}\;\left|x_{i}\right|$	30	${\left[\begin{array}{c} {}*20c-10,10 \end{array}\right]}^{n}$	0
$F_{2}\left(x\right)=\sum_{i=1}^{n}\;{\left(\sum_{j=1}^{i}\;x_{j}\right)}^{2}$	30	${\left[\begin{array}{c} {}*20c-100,100 \end{array}\right]}^{n}$	0
$F_{3}\left(x\right)=\underset{i}{max}\;\left\{\left|x_{i}\right|,1\leq i\leq n\right\}$	30	${\left[\begin{array}{c} {}*20c-100,100 \end{array}\right]}^{n}$	0
$F_{4}\left(x\right)=\sum_{i=1}^{n}\;ix_{i}^{\text{4}}+random\left[0,1\right)$	30	${\left[\begin{array}{c} {}*20c-1.28,1.28 \end{array}\right]}^{n}$	0
Multimodal function	Dimension	Upper and lower bounds	Optimal solution
$F_{5}\left(x\right)=\sum_{i=1}^{n}\;-x_{i}sin\left(\begin{array}{c} {}*20c\sqrt{\left|x_{i}\right|} \end{array}\right)$	30	${\left[\begin{array}{c} {}*20c-500,500 \end{array}\right]}^{n}$	−418.9829n
$F_{6}\left(x\right)=\sum_{i=1}^{n}\;\left[x_{i}^{2}-10cos\left(2\pi x_{i}\right)+10\right]$	30	${\left[\begin{array}{c} {}*20c-5.12,5.12 \end{array}\right]}^{n}$	0

**Table 7:** Algorithm parameter settings.

Algorithm	Parameters				
GWO	N = 50	T = 500	D = 100	ub = 100	lb = −100
PSO	N = 50	T = 500	D = 100	ub = 100	lb = −100
WOA	N = 50	T = 500	D = 100	ub = 100	lb = −100
ABC	N = 50	T = 500	D = 100	ub = 100	lb = −100
SSA	N = 50	T = 500	D = 100	ub = 100	lb = −100
ISSA	N = 50	T = 500	D = 100	ub = 100	lb = −100

We employed the Root Mean Square Error (RMSE) as the fitness function (Fitness), as shown in Eq. (19). All weights and biases to be optimized in the BP neural network are represented in vector form as *θ*. For a given *θ*, the network’s predicted value for *x*ᵢ is denoted as *ŷᵢ*(*θ*).


$$Fitness(\theta )=RMSE(\theta )=\sqrt{\frac{\text{1}}{n}\sum_{i=1}^{n}\;{\left({\hat{y}}_{i}(\theta )-y_{i}\right)}^{2}}$$ (19)

where n is the number of samples, *x*ᵢ represents the input feature vector (including *Y*, *R*, *Q*, and *EI*), *y*_*i*_ denotes the target output (E). *y*ᵢ is the true value, *ŷᵢ* is the predicted value.

This function maintains the same units as the target variable *E*, providing an intuitive measure of the model’s prediction error. During each iteration of the ISSA algorithm, the *θ* vector of each sparrow individual in the “population” is input into this formula to obtain the corresponding Fitness value, with the algorithm’s objective being to minimize this value.

**Fig 4:**
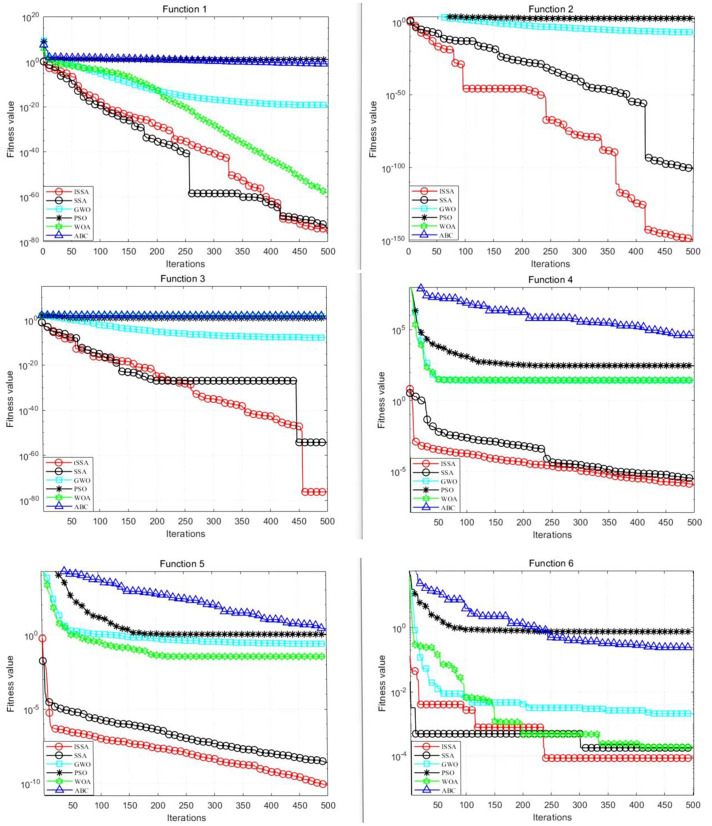
Fitness curves of the 6 test functions.

The fitness curves for the ISSA and the other optimization algorithms across the six test functions are illustrated in Fig 4, with the results derived from 500 iterations. For both unimodal and multimodal test functions, the ISSA consistently outperforms the other five algorithms, achieving the highest fitness levels. This performance enhancement indicates that the integration of tent mapping and adaptive t-distribution mutation significantly bolsters both the local and global optimization capabilities. Compared with the SSA and the other algorithms, the ISSA results in marked improvements in convergence performance and speed.

In contrast to PSO, whose velocity‐position update often leads to premature convergence in complex landscapes, and GWO, which, despite its simple *α/β/δ* encircling mechanism, exhibits slow convergence in later iterations, WOA’s alternating bubble‐net hunting and spiral updating can suffer from cyclic stagnation, and ABC’s scout–employed abandonment strategy, while flexible, yields relatively low search efficiency. ISSA overcomes these limitations by using Tent mapping to generate a uniformly diverse initial population and by introducing an adaptive t-distribution mutation that applies iteration-dependent perturbations to escape local traps. As a result, ISSA achieves stronger global search capability, faster convergence, and greater stability compared to these classical algorithms.

### 3.5. Analysis of CO_2_ emission prediction results for civil aviation

To assess the accuracy of the ISSA-BP model in predicting CO_2_ emissions from civil aviation, a systematic comparative analysis is conducted among BP models optimized by various algorithms. This analysis involves a comparison of the ISSA-BP model with several classic algorithm-optimized BP models, including GWO-BP, PSO-BP, WOA-BP, ABC-BP, and SSA-BP. This comparative framework effectively highlights the predictive advantages of the ISSA-BP model in estimating civil aviation CO_2_ emissions. Additionally, the ISSA-BP model is analyzed alongside traditional BP models, a support vector machine (SVM) model, and the ISSA-SVM model, further clarifying its superiority in this context.

The parameter settings of the BP neural network are shown in Table 8.

**Table 8:** Parameter settings of the BP network.

Parameter	Explanation	Value
Inputnum	Number of input layer nodes	4
Outputnum	Number of output layer nodes	1
Hiddennum	Number of hidden layer nodes	13
Net.trainParam.epochs	Maximum number of iterations for training	1000
Net.trainParam.lr	Network learning rate	0.01
Net.trainParam.goal	Minimum error of the training target	1e-4
Random_state	Random number seed	1

Table 8 presents the parameter indicators of the BP neural network utilized in this study. The configuration includes four input layer nodes, one output layer node, and thirteen hidden layer nodes. The maximum number of iterations for training is set to 1000, with a network learning rate of 0.01. The minimum error of the training target is 1e-4, and the fixed random number seed is 1. The training function employed is trainlm, and leungdm serves as the adaptive learning function. The performance function is defined as the mean squared error (MSE), and the activation function is a sigmoid function.

#### 3.5.1. Comparative analysis of multiple algorithm optimization models.

In this study, the dataset is applied to six models—GWO-BP, PSO-BP, WOA-BP, ABC-BP, SSA-BP, and ISSA-BP—for comparative analysis. To ensure fairness and consistency in evaluation, all the models employ identical network parameter settings. A summary of the evaluation indicators for each model is presented in Table 9.

**Table 9:** Comparison of the results of optimizing BP with different algorithms.

Model	R^2^	MAE	RMSE	MSE	MAPE (%)
ISSA-BP	**0.9996**	**0.1262**	**0.1823**	**0.0332**	**0.7000**
GWO-BP	0.9988	0.2015	0.2984	0.0890	1.2800
WOA-BP	0.9970	0.4056	0.4748	0.2254	4.5700
PSO-BP	0.9971	0.3224	0.4675	0.2186	1.9000
ABC-BP	0.9961	0.3987	0.2012	0.0405	4.9600
SSA-BP	0.9995	0.1611	0.1883	0.0354	2.1000

The results in Table 9 reveal that the ISSA-BP model excels across all indicators. Notably, its R² value (0.9996 significantly surpasses those of the other models, indicating exceptional fitting capability. Furthermore, the model’s MAE, RMSE, MSE, and MAPE are 0.12617, 0.18226, 0.0332, and 0.00698, respectively—the lowest among all the models. These findings suggest that the ISSA-BP model offers substantial advantages over BP models optimized with alternative algorithms in predicting civil aviation CO_2_ emissions. This superior performance can be attributed to enhancements in the ISSA, which effectively mitigates the issue of convergence to local optima when optimizing the BP neural network parameters, thereby improving the model’s global search capabilities and convergence speed.

In contrast, while the other optimization algorithms do improve the BP model’s performance to some extent, they still fall short of matching the predictive accuracy of the ISSA-BP model in handling complex datasets.

Computational efficiency and model complexity (Table 10) demonstrates that the ISSA-BP model exhibits significant efficiency advantages while maintaining prediction accuracy. In terms of convergence speed, the total convergence time for ISSA-BP is 114.1 seconds (108.2s optimization and 5.9s training), representing a 23.8% improvement over conventional SSA-BP. Compared to other algorithms, ISSA-BP’s optimization phase consumes only 58.1% of PSO-BP’s time and 36.7% of ABC-BP’s time. This acceleration stems from ISSA’s enhanced mechanism that reduces invalid search iterations. Regarding complexity optimization, All comparative models employ the unified 4-13-1 network architecture with 79 total parameters (65 weights and 14 biases). ISSA-BP achieves the optimal parameter-to-performance ratio. Notably, while models like GWO-BP and WOA-BP show slightly shorter training times, their extended optimization phases result in lower overall efficiency.

**Table 10:** Computational efficiency and model complexity.

Model	Time required for convergence (s)			Model complexity			
	Total	Optimization algorithm	Network training	network structure	Total parameter	weight	bias
ISSA-BP	114.1	108.2	5.9	4-13-1	79	65	14
GWO-BP	146.47	141.55	4.92				
WOA-BP	158.75	153.2	5.55				
PSO-BP	191.03	186.1	4.93				
ABC-BP	300.78	295.04	5.74				
SSA-BP	155.73	150.73	5				

In summary, the ISSA-BP model demonstrates significant superiority in managing high-dimensional and intricate data. Its high accuracy and robustness render it an effective tool for predicting complex phenomena such as civil aviation CO_2_ emissions, further validating the efficacy of the improved SSA in optimizing BP network parameters.

#### 3.5.2. Comparative analysis of multiple model prediction results.

**Fig 5:**
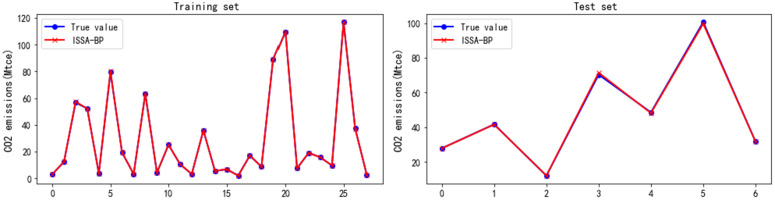
Prediction results for civil aviation CO_2_ emissions obtained via the ISSA-BP model.

**Fig 6:**
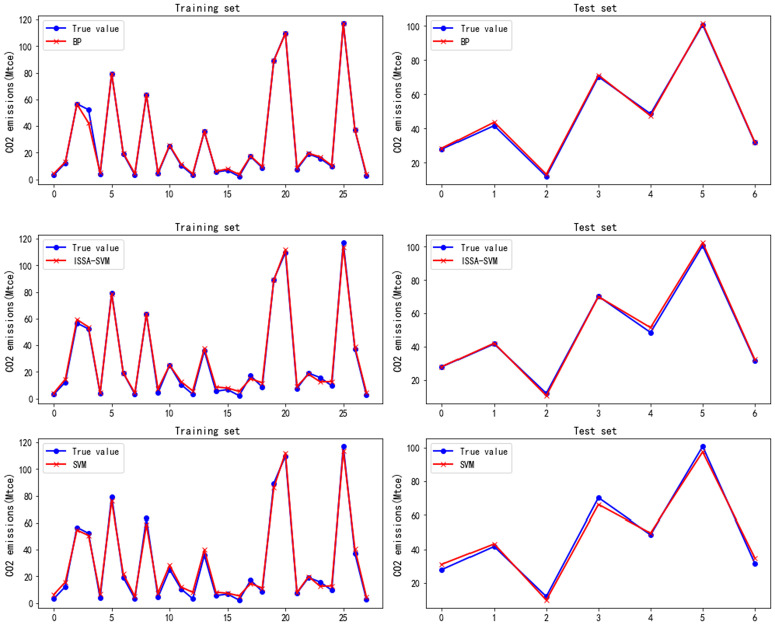
Prediction results for civil aviation CO_2_ emissions obtained via the BP, ISSA-SVM, and SVM models.

By optimizing the parameters of the BP neural network through the ISSA, an ISSA-BP model was developed for forecast CO_2_ emissions in civil aviation. The prediction results from the ISSA-BP model are illustrated in Fig 5. To further corroborate the model’s predictive accuracy, this study also contrasts the CO_2_ emission forecasts derived from the BP, SVM, and ISSA-SVM models, as depicted in Fig 6. A comparison of Figs 5 and 6 reveals that the ISSA-BP model results in a lower prediction error in the estimation of civil aviation CO_2_ emissions.

To comprehensively assess the predictive capabilities of these models, five evaluation metrics are employed to rigorously analyze each model’s performance, further substantiating the superiority of the ISSA-BP model in predicting civil aviation CO_2_ emissions. Tables 11 and 12 present summaries of the quantitative indicators, including R², MAE, RMSE, MSE, and MAPE, for all the prediction models on the training and test datasets, respectively. The results unequivocally indicate that the ISSA-BP model outperforms all the other models across all the metrics, thereby confirming its exceptional overall performance.

**Table 11:** Performance evaluation index results on each model training set.

Model\Error	R^2^	MAE	RMSE	MSE	MAPE (%)
ISSA-BP	0.9999	0.0396	0.0656	0.0043	1.1833
BP	0.9964	0.3097	0.6338	0.4017	13.5610
ISSA-SVM	0.9962	0.5597	0.6520	0.4251	25.7871
SVM	0.9926	0.8373	0.9044	0.8180	34.2694

**Table 12:** Performance evaluation index results on each model test set.

Model\Error	R^2^	MAE	RMSE	MSE	MAPE (%)
ISSA-BP	0.9996	0.1262	0.1823	0.0332	0.6985
BP	0.9983	0.3150	0.3522	0.1240	3.2616
ISSA-SVM	0.9973	0.3362	0.4506	0.2031	3.2720
SVM	0.9897	0.8208	0.8801	0.7745	7.6743

(1) Analysis of the deviation between the model prediction curve and actual values

Figs 5 and 6 illustrate that the prediction curves for each model oscillate around the true values. Notably, the SVM model displays the largest oscillation amplitude, resulting in a substantial deviation between the predicted and actual CO_2_ emissions from civil aviation, thus yielding lower prediction accuracy than the other models do. Although the ISSA-SVM model has been improved on this basis, reducing the deviation from the true value, it still results in considerable errors at certain points. In contrast, the BP model has smaller prediction errors and outperforms both the SVM and its improved models. As shown in Fig 5, the ISSA-BP model achieves the smallest error in forecasting civil aviation CO_2_ emissions, with all the values predicted on its training set aligning closely with the actual values. The prediction results further reveal that, on the test set, the model’s predicted values are almost entirely consistent with the actual values. However, Fig 6 indicates that various discrepancies persist between the values predicted by the BP, SVM, and ISSA-SVM models and the actual values.

(2) Improving model robustness and prediction accuracy through the ISSA optimization algorithm

**Fig 7:**
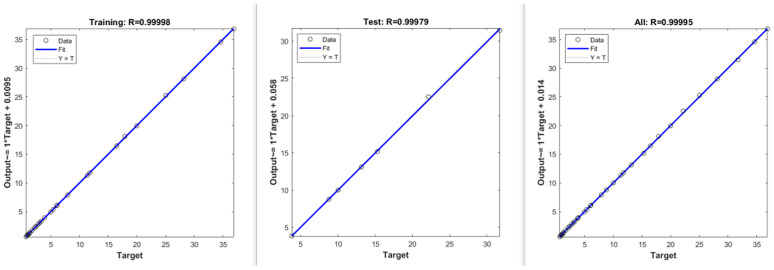
Training set fitting of the ISSA-BP model.

Figs 5 and 6 also demonstrate that the robustness of both the BP and SVM models is markedly enhanced following the integration of the ISSA optimization algorithm. This optimization reduces the fluctuations in the predicted values around the true values, thereby further increasing the prediction accuracy. Notably, the ISSA-BP model’s prediction curve for civil aviation CO_2_ emissions aligns closely with the true values, revealing an exceptional fitting effect, as depicted in Fig 7. The fitting graph indicates that the scatter points are distributed predominantly along the diagonal, with a correlation coefficient approaching 1. Overall, the ISSA-BP model is identified as the most effective in predicting civil aviation CO_2_ emissions, demonstrating significant advantages.

(3) Quantitative comparative analysis of model prediction performance

A comparative analysis of the quantitative indicators reveals the predictive performance of each model, with the ISSA-BP model exhibiting a significantly lower prediction error than its counterparts. Tables 11 and 12 present the performance disparities among the models. Notably, the SVM model demonstrates the poorest performance, with the highest MAE, RMSE, MSE, and MAPE in the test set, indicating a considerable average deviation between the predicted and actual values. In contrast, the BP model outperforms the SVM model and its improved models. Further analysis indicates that the ISSA-BP model excels across all the evaluation metrics, achieving an R^2^ value close to 1 on the test set and MAE, RMSE, MSE, and MAPE values of 0.1262, 0.1823, 0.0332, and 0.6985, respectively, which are substantially lower than those of the other models. This underscores the significant advantages of the ISSA in optimizing the parameters of the BP model, resulting in exceptional performance in predicting civil aviation CO_2_ emissions.

In summary, the ISSA-BP model achieves an R^2^ value near 1 on both the training and test sets, indicating excellent fitting performance on the dataset and markedly superior predictive accuracy compared with the BP, SVM, and ISSA-SVM models. The higher R^2^ value, coupled with lower MAE, RMSE, MSE, and MAPE values, further highlights the model’s robust performance, indicating its adaptability to diverse datasets.

Furthermore, comprehensive evaluation demonstrates that the proposed ISSA-BP model exhibits superior performance across all assessment metrics compared to both benchmark models, as shown in Table 13. In terms of prediction accuracy, the ISSA-BP achieves near-perfect goodness-of-fit (R^2^ = 0.9996), outperforming the linear regression (R^2^ = 0.9991) and VAR models (R^2^ = 0.9973). The model’s MAE (0.1262) represents merely 15.9% of the linear regression model’s error and 9.5% of the VAR model’s, while its RMSE (0.1823) shows significantly reduced prediction error standard deviation. Regarding error control, the ISSA-BP’s MSE (0.0332) is 31 times smaller than linear regression’s and 89 times smaller than VAR’s, with its MAPE (0.7%) maintaining average percentage errors within 1%. These quantitative advantages translate to an 84–85% prediction error reduction compared to traditional linear regression, and particularly notable 91.6% improvement in MAPE over the VAR model.

**Table 13:** Performance evaluation index results on ISSA-BP and econometric models.

Model	R^2^	MAE	RMSE	MSE	MAPE (%)
ISSA-BP	**0.9996**	**0.1262**	**0.1823**	**0.0332**	**0.7**
Linear Regression	0.9991	0.7955	1.0186	1.0376	5.8367
VAR	0.9973	1.3292	1.723	2.9688	8.3185

### 3.6. Statistical test and robustness verification

#### 3.6.1. Endogeneity analysis.

To address potential endogeneity issues in the model, all explanatory variables (transportation revenue, total transport turnover, total transportation volume, and energy intensity) were treated as endogenous. Their first-period lagged values were used as instrumental variables in a two-stage least squares (2SLS) estimation. This approach is justified based on the following considerations:

Instrument Validity: The lagged variables are highly correlated with their current counterparts but are not influenced by contemporaneous error terms.

Overidentification Test: The number of instrumental variables (4) equals the number of endogenous variables, resulting in an exactly identified model.

Model Specification: The F-statistics from the first-stage regression all exceed 10, ruling out concerns regarding weak instruments.

Table 14 reveals the 2SLS estimation results, confirming the presence of endogeneity. After controlling for endogeneity, total transport turnover shows a significant negative effect (p < 0.01), indicating that improving operational efficiency can effectively reduce carbon emissions. In contrast, total transportation volume retains a significant positive impact, underscoring the environmental cost associated with the expansion of the aviation industry. The influence of energy intensity becomes statistically insignificant, suggesting that technological improvements alone have limited emission reduction effects. Additionally, the effect of transportation revenue turns insignificant, implying a possible decoupling between economic growth and carbon emissions.

**Table 14:** Endogeneity Test Results (2SLS Estimation).

Variable	Coefficient	Std. Error	t-value	p-value
lnY	−0.208	0.23	−0.9	0.373
lnR	−0.788***	0.263	−2.99	0.006
lnQ	2.152***	0.425	5.07	0
lnEI	0.322	2.378	0.14	0.893

Note: ***, **, and * denote significance at the 1%, 5%, and 10% levels, respectively.

#### 3.6.2. Diebold-Mariano test.

The Diebold Mariano test is used to test whether there is a significant difference in the accuracy of the test set data of the two prediction models.

(1) Statement of statistical significance

The Diebold Mariano test results are shown in Table 15, the prediction performance difference between ISSA-BP model and all comparison models (BP, SVM, ISSA-SVM) reached statistical significance level (p < 0.05), which confirmed the effectiveness of the improved method proposed in this study.

**Table 15:** Diebold-Mariano tests results of model prediction performance.

Model comparison	DM statistics	p value	Effect direction
ISSA-BP vs BP	72.0245**	<0.01	ISSA-BP
ISSA-BP vs SVM	124.0762**	<0.01	ISSA-BP
ISSA-BP vs ISSA-SVM	4.0857**	0.0065	ISSA-BP

Note: ** indicates p < 0.01

(2) Difference degree analysis

The size of DM statistics further reveals the degree of difference. Compared with traditional SVM, ISSA-BP has the most significant improvement (DM = 124.08), followed by BP neural network (DM = 72.02). Even compared with ISSA-SVM which also uses the improved sparrow algorithm, ISSA-BP still shows significant advantages (DM = 4.09, P = 0.0065), which shows that the optimization effect of BP neural network framework combined with sparrow algorithm is better than that of SVM framework.

#### 3.6.3. Robustness testing and sensitivity analysis.

To ensure model reliability, this study adopted a two-layer validation framework. Control variable tests demonstrate that the model maintains stable prediction performance when key parameters vary within reasonable ranges. The results are shown in Table 16.

**Table 16:** Sensitivity Analysis results of key parameters.

Parameter	Test range	Optimal value	MSE fluctuation range
Hidden nodes	7-15	13	±0.5
Sparrow population size	20-100	50	±1
t-distribution mutation probability	0.1-0.9	0.5	±0.3
Discoverer proportion	0.1-0.4	0.2	±1.1
Maximum iterations	50-300	100	±1.2
Warning Threshold	0.5-0.9	0.6	±0.6

(1) Model architecture sensitivity

Three neural network architectures (4-7-1, 4-13-1, and 4-15-1) were tested. The results confirm that the 4-13-1 architecture remains optimal, validating the rationality of the original structure.

(2) Parameter sensitivity

A grid search was performed on five key parameters of the ISSA algorithm (sparrow population size and t-distribution mutation probability).

The results demonstrate that our improved ISSA maintains stable performance across all tested parameter variations, with MSE fluctuations remaining within ±1.2% of optimal values. This comprehensive analysis confirms the robustness of our modifications to population initialization and mutation operations under diverse parameter settings.

### 3.7. Uncertainty analysis

#### 3.7.1. Neural network cross validation.

Given the limited sample size characteristic of civil aviation CO₂ emission prediction, this study employed five-fold cross-validation to evaluate the ISSA-BPNN model’s performance, thereby reducing the randomness associated with single trial predictions. This methodology partitions the dataset into five mutually exclusive subsets, ensuring each sample participates in both training and testing processes, effectively mitigating limitations caused by small sample sizes. As presented in Table 17, the ISSA-BPNN model demonstrated excellent predictive performance in cross-validation: achieving an average accuracy of 90.54% with a root mean square error (RMSE) of 0.3918 million tons, indicating consistent prediction precision across different data partitions. Furthermore, the model’s coefficient of determination (R) reached 0.9966, approaching the ideal value of 1, further confirming strong correlation between predicted and actual values. These results robustly demonstrate that the ISSA-BPNN model possesses superior generalization capability and goodness-of-fit, establishing it as a reliable tool for civil aviation CO₂ emission forecasting.

**Table 17:** Results of 5-fold cross-validation of ISSA-BP.

Fold	Per-fold accuracy (ACC)/%	Per-fold root mean square error (RMSE)/Mt	Per-fold determinable coefficient(R^2^)	Average ACC/%	Average RMSE/Mt	Average R^2^
1	86.03	0.5732	0.9885	90.54	0.3918	0.9966
2	90.72	0.5376	0.9975			
3	90.72	0.1586	0.9999			
4	94.56	0.3853	0.9981			
5	90.69	0.3045	0.9993			

#### 3.7.2. Model performance evaluation and uncertainty analysis.

**Fig 8:**
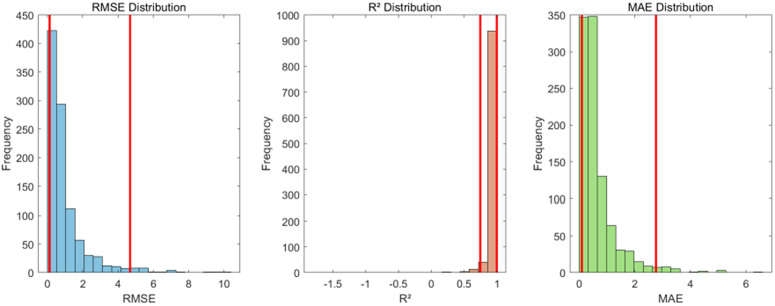
Distributions and 95% confidence intervals (CIs) of RMSE, R², and MAE.

To quantify the uncertainty of the predictive model, this study employed the Bootstrap resampling method (n = 1000) to estimate the distributions and 95% confidence intervals (CIs) of RMSE, R^2^, and MAE. The results are shown in Fig 8.

RMSE: 1.016 (95% CI: 0.127–4.686). The left -skewed distribution (Fig 8, left) indicates that most prediction errors are concentrated in the lower range, though a few high-error outliers exist. R^2^: 0.967 (95% CI: 0.748–1.000). The right -skewed distribution (Fig 8, middle) suggests near-optimal explanatory power (R^2^ ≈ 1) in most cases, with only minor performance degradation in rare scenarios. MAE: 0.669 (95% CI: 0.099–2.763). Similarly, its left -skewed distribution (Fig 8, right) further confirms the model’s robustness.

Therefore, despite some error fluctuations, the model exhibits excellent overall performance (median R^2^ > 0.96).

## 4. Policy implications

Our analysis indicates that reduced energy intensity (EI) significantly curbs aviation CO₂ emissions, primarily driven by technological advances and fleet renewal. The 2SLS estimation further reveals that improving operational efficiency has greater emission-reduction potential than technological measures alone. Additionally, the positive impact of total transportation volume highlights the necessity of establishing growth constraint mechanisms such as carbon emission caps.

To effectively curb aviation CO₂ emissions, a multi-pronged strategy is essential. First, emission-reduction technologies should be promoted by enhancing fuel efficiency through technological innovations and expanding technical training. Second, efficiency-oriented operations must be prioritized—including load factor optimization and route network redesign. Third, scale management mechanisms such as carbon emission caps and growth constraints should be implemented to mitigate the strong positive impact of transport volume on emissions. Fourth, integrated policy systems combining market and regulatory instruments should be developed to address the limited individual significance of emission intensity. Finally, external measures such as forest carbon sinks should be utilized to neutralize residual emissions, supported by global carbon market mechanisms.

In summary, the key to aviation emission reduction lies not in suppressing growth itself, but in transforming growth patterns through efficiency gains, operational optimizations, and well-designed policy instruments. Integrating internal measures with external offsets can accelerate China’s aviation sector toward net-zero by 2050.

## 5. Conclusions

Accurately forecasting civil aviation CO_2_ emissions is essential for assessing and mitigating the environmental impact of the aviation industry. However, carbon emissions from civil aviation are influenced by a multitude of complex factors. Building on previous research, this paper identifies four key indicators: transportation revenue, total civil aviation transportation turnover, total civil aviation transportation volume, and energy intensity. To address the challenges associated with parameter selection in the BP neural network model, this study introduces a civil aviation carbon emission prediction model based on the ISSA-BP framework. Through a comparative analysis with various prediction models, utilizing five evaluation indicators to assess performance comprehensively, the following principal conclusions are drawn:

(1) Performance advantages of the ISSA-BP model: A comparison of the optimized BP neural network model with different algorithms reveals that the ISSA-BP model has superior prediction accuracy. Specifically, the ISSA-BP model achieves an R^2^ value of 0.99956, with an MAE of 0.12617, an RMSE of 0.18226, an MSE of 0.03322, and a MAPE of 0.00698. This model not only excels in terms of prediction accuracy but also exhibits remarkable stability and robustness.
(2) Results of the model comparison analysis: Among four models—ISSA-BP, BP, ISSA-SVM, and SVM—the ISSA-BP model consistently outperforms the others on both the training and test sets. It presents a higher R^2^ value and lower MAE, RMSE, MSE, and MAPE, indicating excellent fitting performance with minimal error, thereby further demonstrating the advantages of the ISSA-BP model in predicting civil aviation CO_2_ emissions.
(3) Practical application value of the research: The ISSA-BP model developed in this study not only serves as an effective tool for the precise prediction of CO_2_ emissions in civil aviation but also offers a scientific basis for policy-makers for the formulation and implementation of CO_2_ reduction strategies. This model aids in the understanding and quantification of the driving factors behind CO_2_ emissions, facilitating the proposal of more targeted environmental protection measures.

Despite the progress made in constructing and optimizing CO_2_ emission prediction models, several limitations remain:

(1) Indicator dimensions: This study addresses primarily macrolevel factors that influence civil aviation CO_2_ emissions. However, microlevel variables—such as the emission characteristics of specific routes and aircraft types—could yield more nuanced predictions. Future research should aim to refine these microlevel indicators.
(2) Optimization and improvement of the ISSA-BP model: While some advancements have been achieved in optimizing the traditional SSA, the inherent complexity and limitations of the algorithm leave room for further improvement of the global search capabilities and convergence speed. Future investigations could explore additional improvement avenues, such as integrating diverse heuristic strategies or introducing varied search mechanisms, to further enhance the optimization performance of BP neural networks.

## Supporting information

S1 Data Minimal data set. (XLSX)

## References

[1] Romanello M, Whitmee S, Mulcahy E, Costello A. Further delays in tackling greenhouse gas emissions at COP28 will be an act of negligence. Lancet. 2023;402(10417):2055–7. doi: 10.1016/S0140-6736(23)02584-9 38006898

[2] Brasseur GP, Gupta M. Impact of aviation on climate: research priorities. Bull Am Meteorol Soc. 2010;91(4):461–4.

[3] Zhang F, Graham DJ. Air transport and economic growth: a review of the impact mechanism and causal relationships. Trans Rev. 2020;40(4):506–28. doi: 10.1080/01441647.2020.1738587

[4] Comac. Market Forecast Annual Report: 2024-2043. 2023. Available from: http://www.comac.cc/fujian/2024-2043nianbao_en.pdf

[5] Benchimol J, El-Shagi M. Forecast performance in times of terrorism. Economic Modelling. 2020;91:386–402. doi: 10.1016/j.econmod.2020.05.018

[6] Zhu JL, Hu R, Zhang JF. Research on the Measurement and Evolution Characteristics of Aircraft Carbon Emissions in China. Journal of Wuhan University of Technology (Transportation Science & Engineering). 2020;44(3):558–63.

[7] Liu X, Hang Y, Wang Q, Zhou D. Drivers of civil aviation carbon emission change: A two-stage efficiency-oriented decomposition approach. Transportation Research Part D: Transport and Environment. 2020;89:102612. doi: 10.1016/j.trd.2020.102612

[8] Kito M, Nagashima F, Kagawa S, Nansai K. Drivers of CO2 emissions in international aviation: the case of Japan. Environ Res Lett. 2020;15(10):104036. doi: 10.1088/1748-9326/ab9e9b

[9] Xu JH, Wang K. Medium- and long-term carbon emission forecast and technological emission reduction potential analysis China’s civil aviation industry. China Environ Sci. 2022;42(7):3412–24.

[10] Mullainathan S, Spiess J. Machine Learning: An Applied Econometric Approach. J Econ Perspect. 2017;31(2):87–106. doi: 10.1257/jep.31.2.87

[11] Liu Z, Guan D, Wei W, Davis SJ, Ciais P, Bai J, et al. Reduced carbon emission estimates from fossil fuel combustion and cement production in China. Nature. 2015;524(7565):335–8. doi: 10.1038/nature14677 26289204

[12] Aldy JE. Per Capita Carbon Dioxide Emissions: Convergence or Divergence? Environ Resourc Econ. 2006;33(4):533–55. doi: 10.1007/s10640-005-6160-x

[13] Barkan O, Benchimol J, Caspi I, Cohen E, Hammer A, Koenigstein N. Forecasting CPI inflation components with Hierarchical Recurrent Neural Networks. Int J Forecast. 2023;39(3):1145–62. doi: 10.1016/j.ijforecast.2022.04.009

[14] Liu X, Hang Y, Wang Q, Zhou D. Flying into the future: A scenario-based analysis of carbon emissions from China’s civil aviation. J Air Trans Manag. 2020;85:101793. doi: 10.1016/j.jairtraman.2020.101793

[15] Chao H, Agusdinata DB, DeLaurentis D, Stechel EB. Carbon offsetting and reduction scheme with sustainable aviation fuel options: Fleet-level carbon emissions impacts for U.S. airlines. Transport Res Part D Trans Environ. 2019;75:42–56. doi: 10.1016/j.trd.2019.08.015

[16] Yu J, Shao C, Xue C, Hu H. China’s aircraft-related CO2 emissions: Decomposition analysis, decoupling status, and future trends. Energy Policy. 2020;138:111215. doi: 10.1016/j.enpol.2019.111215

[17] Yang H, O’Connell JF. Short-term carbon emissions forecast for aviation industry in Shanghai. J Clean Product. 2020;275:122734. doi: 10.1016/j.jclepro.2020.122734

[18] Brons M, Pels E, Nijkamp P, Rietveld P. Price elasticities of demand for passenger air travel: a meta-analysis. J Air Transp Manag. 2002;8(3):165–75. doi: 10.1016/s0969-6997(01)00050-3

[19] Yang L, Hu Y-J, Wang H, Li C, Tang B-J, Wang B, et al. Uncertainty quantification of CO2 emissions from China’s civil aviation industry to 2050. J Environ Manage. 2023;336:117624. doi: 10.1016/j.jenvman.2023.117624 36868152

[20] Wang Q, Li S, Li R, Jiang F. Underestimated impact of the COVID-19 on carbon emission reduction in developing countries - A novel assessment based on scenario analysis. Environ Res. 2022;204(Pt A):111990. doi: 10.1016/j.envres.2021.111990 34481817 PMC9749383

[21] Zhao X, Han M, Ding L, Calin AC. Forecasting carbon dioxide emissions based on a hybrid of mixed data sampling regression model and back propagation neural network in the USA. Environ Sci Pollut Res Int. 2018;25(3):2899–910. doi: 10.1007/s11356-017-0642-6 29143932

[22] Deschênes O, Greenstone M. The Economic Impacts of Climate Change: Evidence from Agricultural Output and Random Fluctuations in Weather. Am Econ Rev. 2007;97(1):354–85. doi: 10.1257/aer.97.1.354

[23] Rumelhart DE, Hinton GE, Williams RJ. Learning representations by back-propagating errors. 1986.

[24] Deng Y, Zhou X, Shen J, Xiao G, Hong H, Lin H, et al. New methods based on back propagation (BP) and radial basis function (RBF) artificial neural networks (ANNs) for predicting the occurrence of haloketones in tap water. Sci Total Environ. 2021;772:145534. doi: 10.1016/j.scitotenv.2021.145534 33571763

[25] Wu D, Zhang D, Liu S, Jin Z, Chowwanonthapunya T, Gao J, et al. Prediction of polycarbonate degradation in natural atmospheric environment of China based on BP-ANN model with screened environmental factors. Chem Eng J. 2020;399:125878. doi: 10.1016/j.cej.2020.125878

[26] Li X, Cheng X, Wu W, Wang Q, Tong Z, Zhang X, et al. Forecasting of bioaerosol concentration by a Back Propagation neural network model. Sci Total Environ. 2020;698:134315. doi: 10.1016/j.scitotenv.2019.134315 31783453

[27] Xue J, Shen B. A novel swarm intelligence optimization approach: sparrow search algorithm. Syst Sci Cont Eng. 2020;8(1):22–34. doi: 10.1080/21642583.2019.1708830

[28] Yang X, Liu J, Liu Y, et al. A novel adaptive sparrow search algorithm based on chaotic mapping and t-distribution mutation. 2021.

[29] Wang Z, Liu Y, Song R. A Crack Image Segmentation Algorithm Based on Adaptive T-distribution[C/OL]. 2021 China Automation Congress (CAC). Beijing: IEEE; 2021, p. 3078–83 [2024-05-29].

[30] Lan KT, Lan CH. Notes on the distinction of Gaussian and Cauchy mutations. In: 2008 Eighth International Conference on Intelligent Systems Design and Applications: Volume 1. 2008, p. 272–7.

[31] Hu M, Zhu L. A novel fractional multivariate GM(1,N) model with interaction effects and its application in forecasting carbon emissions from China’s civil aviation. Grey Syst Theor Appl. 2023;13(3):612–28.

[32] Zhou W, Wang T, Yu Y, Chen D, Zhu B. Scenario analysis of CO2 emissions from China’s civil aviation industry through 2030. Appl Energy. 2016;175:100–8. doi: 10.1016/j.apenergy.2016.05.004

[33] Ipcc, I.P.C.C. 2006 IPCC Guidelines for National Greenhouse Gas Inventories. 2006. Available from: https://www.osti.gov/etdeweb/biblio/20880391

[34] Bakay MS, Ağbulut Ü. Electricity production based forecasting of greenhouse gas emissions in Turkey with deep learning, support vector machine and artificial neural network algorithms. J Clean Produc. 2021;285:125324. doi: 10.1016/j.jclepro.2020.125324

[35] Wang K, Li K, Du F, Zhang X, Wang Y, Sun J. Research on prediction model of coal spontaneous combustion temperature based on SSA-CNN. Energy. 2024;290:130158. doi: 10.1016/j.energy.2023.130158

[36] Dai Y, Zhao P. A hybrid load forecasting model based on support vector machine with intelligent methods for feature selection and parameter optimization. Appl Energy. 2020;279:115332. doi: 10.1016/j.apenergy.2020.115332

[37] Jianhua L, Zhiheng W. A Hybrid Sparrow Search Algorithm Based on Constructing Similarity. IEEE Access. 2021;9:117581–95. doi: 10.1109/access.2021.3106269

[38] Zhang C, Ding S. A stochastic configuration network based on chaotic sparrow search algorithm. Knowl-Bas Syst. 2021;220:106924. doi: 10.1016/j.knosys.2021.106924

[39] Wang H, Wu X, Gholinia F. Forecasting hydropower generation by GFDL-CM3 climate model and hybrid hydrological-elman neural network model based on improved sparrow search algorithm (ISSA). Concurr Comput: Pract Exp. 2021;33(24):e6476.

